# Knowledge, attitudes, and practices regarding antimicrobial resistance and antimicrobial stewardship among healthcare workers in outpatient medical centers in Kenya: a qualitative study

**DOI:** 10.1017/ash.2025.41

**Published:** 2025-05-19

**Authors:** Mary W. Kaniu, Wahu R. Gitaka, Rupali Jain, Ann N. Munyare, Rodney D. Adam, Aliza Monroe-Wise

**Affiliations:** 1 Department of Paediatrics and Child Health, Aga Khan University East Africa Medical College, Nairobi, Kenya; 2 School of Pharmacy, University of Washington, Seattle, WA, USA; 3 Departments of Pathology and Medicine, Aga Khan University East Africa Medical College, Nairobi, Kenya; 4 Division of Allergy and Infectious Diseases, Department of Medicine, University of Washington, Seattle, WA, USA; 5 Department of Pathology, Aga Khan University East Africa Medical College, Nairobi, Kenya; 6 Department of Global Health, University of Washington, Seattle, WA, USA

## Abstract

**Objective:**

Antimicrobial resistance (AMR) is a major global health threat with a projected 10 million deaths globally by 2050. Antimicrobial stewardship (AMS) programs are coordinated efforts involving doctors, nurses, pharmacists, and other healthcare providers. Understanding knowledge, attitudes, and practices of health providers is pivotal for implementing strategies to curb the spread of AMR. The aim of this study was to evaluate knowledge, attitudes, and practices surrounding AMR and AMS among healthcare workers in Kenya.

**Design:**

A qualitative study using in-depth semi-structured interviews

**Setting:**

12 private outpatient clinics in Kenya.

**Participants:**

Healthcare workers including administrators and clinicians.

**Methods:**

A thematic analysis approach was used; the Capability, Opportunity, and Motivation for Behavior model was used to understand the knowledge, attitudes, and practices surrounding AMR and AMS.

**Results:**

Twenty-four participants were interviewed. They had some knowledge regarding AMR but lacked knowledge about AMS and its components. Although participants did not perceive AMR as a problem in their clinics, they reported it was a major problem in the country and globally. There was lack of prioritization of AMS in the clinics.

**Conclusions:**

The lack of knowledge on AMS and its components coupled with failure to recognize AMR as a problem in the facilities led to a lack of prioritization of AMS. There is therefore an urgent need to educate healthcare administrators and clinicians on AMR and AMS to foster a sense of ownership of the problem of AMR and to be pro-active in implementing measures to curb it.

## Introduction

Antimicrobial resistance (AMR) is a major global health threat with a projected 10 million annual deaths globally due to resistant infections by 2050.^1^ In 2019, it was estimated that there were 4.95 million deaths associated with bacterial AMR worldwide with western sub-Saharan Africa (SSA) having the highest all-age death rate attributable to bacterial resistance.^2^ Globally, excessive and indiscriminate use of antimicrobials drives the development of AMR.^3^ Inappropriate antibiotic use is common, particularly in low- and middle-income countries (LMICs) where enforcement of prescribing laws is weak.^4–12^

Antimicrobial stewardship (AMS) is a broad term that refers to comprehensive quality improvement activities and evidence-based knowledge translation strategies to optimize the use of antimicrobial agents, reduce development of resistance to antimicrobials, and improve patient outcomes with a reduction in healthcare costs.^13–16^ AMS programs are interdisciplinary and require the involvement of doctors, nurses, pharmacists, other healthcare providers and support staff. Healthcare workers are key for implementation of AMS programs; therefore, understanding knowledge and attitudes surrounding AMR and AMS is necessary to implement effective AMS programs. Several studies have been conducted globally to assess the knowledge, attitudes, and skills of different cadres of healthcare workers with varying results^17–23^; however, only a few have been carried out in SSA, most of which have been conducted in inpatient settings.^24–26^ Therefore, there is a need to better understand healthcare workers’ experiences and level of understanding of AMR and AMS in outpatient settings in SSA.

This study aims to evaluate the knowledge, attitudes, and practices surrounding AMS and AMR among healthcare workers in Aga Khan University Hospital (AKUH) outpatient medical centers in Kenya.

## Methods

This study was conducted in 12 AKUH outpatient medical centers located in the Nairobi metropolitan area, including centers in Nairobi, Kiambu, and Kajiado counties in Kenya. They were selected based on predetermined criteria, including high patient volumes and availability of permanent medical officers. Medical centers that utilize residency trained doctors (i.e. specialists) were excluded.

The study population included administrators and clinicians working in outpatient health facilities. An administrator was defined as an employee of the facility acting as the facility-in-charge who was either a healthcare worker involved with direct patient care (e.g. nurses, medical officers, laboratory technologists, pharmaceutical technologists, and radiographers) or patient care service coordinators. A clinician was defined as a medical officer who has completed undergraduate medical training and one-year internship and is licensed to practice medicine in Kenya. Purposive sampling was used, and all administrators and doctors working in the selected centers were invited to take part in the study.

Semi-structured in-depth interviews were carried out in English among all participants using an interview guide which was developed by an infectious disease specialist within the study team with experience in conducting AMS training programs and qualitative research. The interview guide was developed using the Capability, Opportunity, Motivation and Behavior (COM- B)^27^ model for behavior change as a framework. Interviews were audiotaped using a handheld recorder and audio files were uploaded onto a secure, password protected, server daily. An experienced transcriber transcribed the audio files into written documents. To ensure accuracy of the transcriptions, a sample of the original audiotapes was compared with the transcribed notes. Transcripts were then uploaded onto a secure, password-protected server. The transcripts were reviewed regularly by two investigators to determine the point at which content saturation was reached.

Data were analyzed using a thematic analysis approach.^28^ Analysis was performed by two investigators (MK and AM), who independently reviewed randomly chosen sections of texts line by line to identify codes that populated an initial codebook. Similar themes were then merged as common concepts, and these were then organized into typologies. A coding schema was created, informed by both a priori themes included in the interview guide, emergent themes, and by the Capability, Opportunity, and Motivation for Behavior (COM-B) framework. Data analysis was iterative whereby themes, subthemes and subcategories were added to reflect variations in the data through weekly meetings. Original transcripts were reviewed using the emergent coding scheme as part of the iterative analytical process to ensure methodological rigor. Any coding concerns were resolved through discussions among the two investigators with further refinement of the codebook parameters.

## Results

### Sample characteristics

Twenty-four participants were enrolled in the study. Of these, 12 were administrators (Table 1). Participants were between 30 and 53 years old, and only three had previously undertaken any formal AMS training. Interviews lasted between 17 and 58 minutes.

**Table 1.** Demographic characteristics

Baseline characteristics (n=24)	Total (%)
Sex	
Male	11 (46%)
Female	13 (54%)
Previous AMR Course	
Yes	3 (13%)
No	21 (87%)
Administrator	
Yes	12 (50%)
No	12 (50%)
Cadre	
Medical Officer	8 (33%)
Other staff	
Nurse	6 (25%)
Pharmaceutical technologist	5 (21%)
Laboratory technologist	3 (13%)
Patient Service Coordinator	1 (4%)
Radiographer	1 (4%)

### Themes

**Figure 1.**
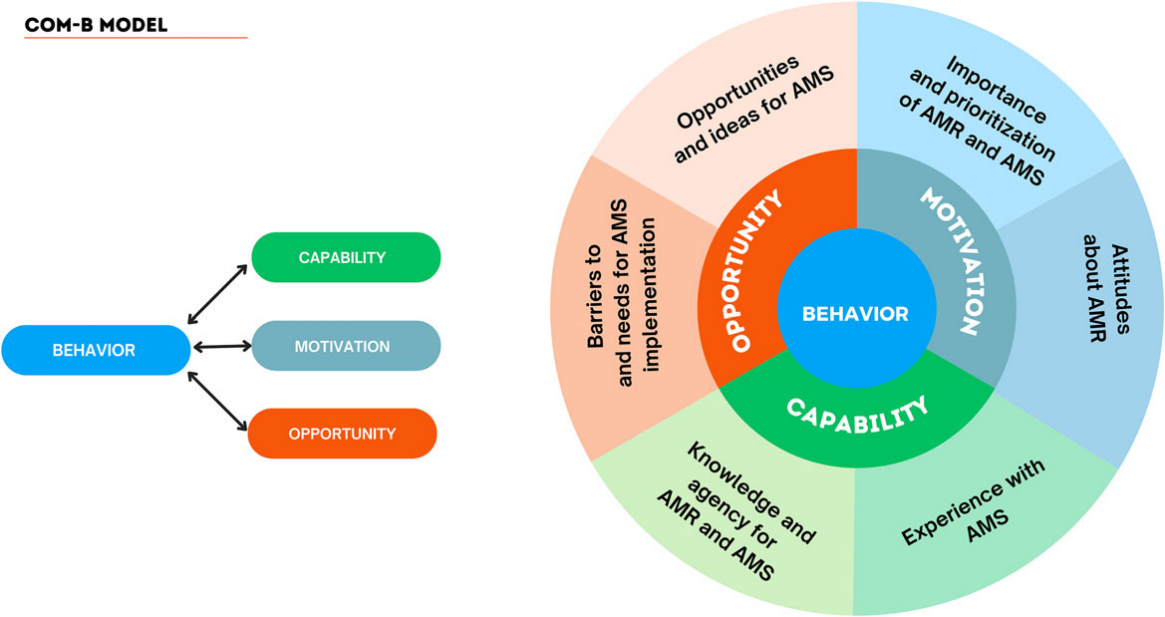
Key Themes under COM-B Model.

The COM-B model was used to better understand the drivers of AMR and barriers to implementation of AMS programs in the study population. The following key themes emerged from the in-depth interviews: knowledge; agency; experience; barriers to and needs for implementation; opportunities and ideas; importance and prioritization; and attitudes. We present the results under the COM-B Framework categories of capability, opportunity, and motivation (Figure 1).

### Knowledge (Capability)

All participants had some understanding of AMR and many of them correctly identified some of its known causes.

*“This is the trend whereby microb[es], be it bacterial, viral, fungal or protozoa, have developed the ability to evade the effects of chemotherapy in the form of antibiotics. The mechanism by which this is evaded is very broad* (*e.g. patients not responding to standard antibiotics”*). KII_001, Medical officer

Some people defined AMR as a person becoming resistant to antibiotics which, while erroneous, does imply having some knowledge of AMR. This erroneous definition was more common than the actual definition of AMR.

Overuse of antibiotics, inappropriate choice and duration of antibiotics, laxity in infection prevention and control (IPC) post COVID-19 and having unqualified healthcare practitioners were some of the factors that participants reported to contribute to AMR.

In contrast to the broad knowledge of AMR, most respondents lacked knowledge of AMS. Some respondents attempted to define AMS, while others had not heard of it or were unsure of its definition. This was evident in both clinicians and administrators. Having specific people in charge of AMS, regulations and policies in place, patient education, prescriber education and judicious antibiotics use were all stated as possible components of AMS.

“*I have not heard the name; this is the first time I am hearing about it from you. But I believe it is something geared towards bringing discipline in drugs and antibiotics usage in the hospitals, in health facilities and in the country.”* KII_011 Other staff

Several knowledge gaps were identified, especially when discussing AMS components and resources required to implement AMS programs. Some participants stated that they had no knowledge at all when it came to AMS.

### Agency (Capability)

Participants quoted several perceived limitations in their agency to affect change in the battle against AMR. These included limitations of cadre, outside prescriptions, and self-prescribing.

“*For us like let’s say now in pharmacy, we might not be able to do much, because they are prescribed by the doctor. So by the time the prescription gets to you, I don’t think you have chances to query. So in my position I would assume that the doctor has examined that patient and has ascertained that there is need to give that particular antibiotic… I think it is only on the doctors, so if they don’t prescribe antibiotics to patients who don’t need antibiotics, then resistance will not occur*.” KII_019 Other staff

Prescriptions written by outside providers, or drugs sold over the counter were also seen as a contributor to AMR but difficult to address in their role. Some people attributed the purchase of over-the-counter antibiotics sold at many pharmacies to patients wanting to avoid high costs of consultation with a clinician, or patients being too busy to wait to be reviewed by a clinician in clinics. COVID-19 was also reported to have exacerbated the problem of self-prescriptions.

### Experience and current AMR practices (Capability)

Some facilities had implemented some strategies to address AMR, such as restricting antibiotic dispensing to patients with an authentic prescription from a qualified medical practitioner. Participants also mentioned that patient and provider education was ongoing, infection prevention measures were in place, and laboratory investigations were being done including ordering blood and urine cultures to guide the need for antibiotics.

*“Yeah we do that on a daily basis because number one we don’t issue antibiotics without prescriptions, so we don’t do over the counter, that is one way of regulating them. And two we counsel on the right usage, compliance, the expected side effects and the importance of completing the dose, to avoid the resistance*.” KII_005 Other staff

### Barriers to and needs for AMS implementation (Opportunity)

Several barriers to implementation of AMS programs were cited. These included human and non-human resources, resistance to change, patient attitudes and practices, pharmaceutical industry influences and perceived lack of central leadership.

Respondents felt that time and human resources were limited and there were not enough employees in the clinics or time to implement AMS while also concentrating on providing patient care. Many respondents stated that patient education was the main AMS activity to be implemented and they lacked time to complete other duties within the clinics, thus limiting time for educating patients.

“*I need human resources, as in people to be able to get information and relay it to the patient. Here we work one person per department and when I call the patient, I may not have ample time to do all this, so we definitely need support, if it is the lab staff, she is all alone, she is here she is there, so in terms of now [patient] education, we may need some people.”* KII_009 Other staff

Lack of access to information, inability to perform cultures, and occasional medication shortages were some of the cited hindrances to AMS. Medication shortages, especially of first line antibiotics, was reported to contribute to use of “higher” or broader classes of antibiotics which was thought to contribute to AMR. Some participants felt that colleagues might be resistant to changing their current prescribing patterns and this would hinder implementation of AMS programs.

Promotion of specific pharmaceutical agents by medical representatives was thought to influence antibiotic prescriptions by doctors and was cited as a barrier to AMS by increasing unnecessary antibiotic prescriptions.

*“I will give you an example; there is a drug called [drug name] owned by [pharmaceutical company], they did a very big launch and sold the product, so you see this is now market driven, so now a lot of people are now shifting to [drug name].”* KII_015 Medical Officer

The participants felt that they lacked adequate guidance from the main hospital and the national authorities, and this was seen as a barrier to implementing AMS programs.

### Opportunities and ideas (Opportunity)

The participants provided ideas on how to address AMR and implement AMS programs in outpatient centers. Education for both patients and healthcare providers was seen as a way of addressing AMR and offering training incentives was suggested as one way of motivating people to undertake further training on AMR and AMS.

The government was seen as a pivotal partner in the implementation of AMS programs. Its involvement was proposed through many activities, including policy development, putting stricter regulations in place, and providing education to both healthcare providers and the public.

*“Let the policy makers come on board,… let the policy makers come up and make serious policies, that are going to help us address this, because if we are going to just talk about it on the ground, and on top there nothing is happening, it is going to be very difficult…If it means going to the government and becoming an act of parliament, so be it.”* KII_011 Other staff

Training healthcare providers consistently and over a long period of time, was also reported as one way of raising awareness about AMR. It was suggested that training should be mandated at regular intervals by employers for sustained AMR education. This would ensure that all healthcare workers are trained and rigorously assessed on their understanding of AMR and AMS prior to starting jobs and have routine re-training time lines to ensure that their knowledge of AMR and AMS is up to date.

Having frequent and consistent reminders for healthcare workers about AMR and AMS was also suggested. Reminders would provide passive education to healthcare providers during normal working hours, contributing to increasing awareness of AMR.

*“Just a reminder on someone’s desk, or maybe it pops up on a screen somewhere, calendar or leaflet. I think it will help a bit. Something to remind you.”* KII_005 Other staff.

Conducting more research on AMR and documenting resistance patterns in Kenya was also proposed as a way of curbing AMR.

### Importance and Prioritization (Motivation)

Nearly all respondents reported that AMR was a serious global threat. Participants mentioned various global ramifications of AMR, including the loss of antibiotic efficacy for future generations, increased cost of healthcare, and morbidity and mortality associated with resistant organisms.

“*I think it is very important to fight AMR because if we lose the ability to fight simple infections then we will have an epidemic on our hands.*” KII_001 Medical officer

However, despite strongly expressed views regarding AMR as a global threat, many participants didn’t perceive AMR as a problem in their clinics and therefore were not prioritizing it in their work.

“*No, I don’t think that antimicrobial resistance is a problem in this clinic.”* KII_003 Other Staff

Most respondents felt that AMR was a problem primarily in outpatient facilities as this is usually the first point of contact for patients before they are sent for inpatient care. It was postulated that, in inpatient settings, laboratory investigations are used to guide antibiotic choice, which are then monitored carefully, hence reducing inappropriate antibiotic use which was not the case in outpatient settings.

### Attitudes (Motivation)

Participants expressed motivation to implement AMS programs, stating that they didn’t think it would be difficult if people understood its importance, given that clinicians are professionals who follow protocols.

***“**I don’t think it would be difficult, to be honest because clinicians usually we are professional people, so we do things according to how they should be done. So, it will be OK.”* KII_023 Medical Officer

Many respondents reported that they would be willing to implement AMS programs in the clinics. Availability of resources and the ability to provide quality and comprehensive services to the patients were some of the factors that were noted to affect the willingness to implement AMS programs.

## Discussion

This study found that providers in outpatient medical centers in Kenya had some understanding of AMR but lacked general knowledge of AMS. While participants felt strongly that AMR was a serious national and international threat, they didn’t perceive it as a local problem despite AMR being considered primarily an outpatient problem. This in turn led to lack of prioritization of AMS in the facilities.

Previous studies have reported both that clinicians and pharmacists have good knowledge of AMR^29,30^ and knowledge of AMS is lacking.^19,31,32^ Poor knowledge of AMS may be partially due to the term “antimicrobial stewardship” being new, first appearing in literature in 1996,^33^ and its definitions have evolved over time. Additionally, AMS programs have been primarily an inpatient activity. There is also no globally accepted and implemented curriculum in undergraduate/postgraduate medical training programs addressing AMS, contributing to the lack of knowledge.^34^ WHO developed an AMS implementation training kit for LMICs which recommends incorporation of AMS training as part of pre-service training.^35^ Implementation of this recommendation would be a good starting point in training both clinicians and administrators on AMS.

Some respondents, especially administrators who held leadership positions, reported feeling limited in their capacity to affect change when it came to addressing AMR since they had limited interaction with patients. In contrast to these findings, pharmacists in Malaysia perceived themselves as playing a central role in implementation of AMS strategies, describing themselves as antimicrobial guardians and antimicrobial advisors.^36^ WHO recommends that AMS teams in LMICs should comprise a prescribing clinician, a pharmacist, a nurse and a (clinical) microbiologist or laboratory technologist in facilities with a microbiology laboratory.^35^ In keeping with this recommendation, more work is needed to empower pharmacists, pharmaceutical technologists, and laboratory technologists working in outpatient clinics on the roles they can undertake in AMS practices. Additionally, clinical microbiology laboratories play a crucial role in AMS^37^ and it is important for laboratory technologists working in outpatient clinics to be involved in AMS programs and to better understand diagnostic components of AMS.

Several factors were cited as barriers to implementation of AMS programs. Lack of human and non-human resources, including lack of access to information; patient attitudes and practices, resistance to change, pharmaceutical influence, and a perceived lack of central leadership were the main barriers reported in this study. While many of these barriers have been reported in previous studies,^38^ the findings of pharmaceutical influence and perceived lack of central leadership were unique to this setting.

Respondents in this study reported contradictory attitudes about AMR: while it was perceived to be a significant threat in the country and globally, most participants didn’t think it was a problem in their centers. Similar contradictory attitudes about AMS have been found in other settings.^39^ This paradoxical mentality among healthcare providers could play a role in ongoing excessive antibiotic use in low-resource settings. In contrast to these findings, physicians in Indonesia reported that in addition to AMR being a global problem, it was also a significant problem in their hospital^40^ but did not report antibiotic overuse in their hospital as a reason for the high burden of AMR. This view of AMR being an external problem is a potential hindrance to the fight against AMR. While many of the participants attributed their lack of prioritization of AMS in their settings to time restraints, the lack of prioritization of AMS in the medical centers could also have resulted from the perception that AMR is not a problem in their clinics. One limitation of this study is that it was conducted in private outpatient centers, the findings may not be generalizable to the entire population of outpatient medical centers; therefore, further studies should be carried out in public outpatient facilities. Participants who were interviewed in this study were not necessarily the decision makers for policy change for AMR/AMS; however, our findings are still instructive regarding general levels of knowledge and widely held attitudes surrounding AMR and AMS. In view of this, we recommend urgent implementation of education programs for both qualified healthcare workers and those in pre-service training to ensure that all healthcare workers, including those that serve as administrators, have a baseline knowledge of AMS on which to build once they start working. Additionally, active sensitization of healthcare workers on AMR and AMS should be undertaken with the government leading this initiative, cascading down to all health facilities with regular assessments to ensure compliance to AMR and AMS guidelines.

In conclusion, while basic knowledge of AMR was commonplace among healthcare providers and administrators in private outpatient facilities in Kenya, knowledge of AMS was far less prevalent, and many providers felt disempowered to act against AMR. Training and team building for AMS could address these issues. Further, participants expressed contradictory attitudes regarding the importance of AMR on a global scale, while minimizing its importance on a local scale. Strong government-level and health system-level leadership and regulatory programmes for AMS may improve health providers’ sense of ownership and responsibility for curbing AMR.

## Supporting information

Kaniu et al. supplementary material 1 Kaniu et al. supplementary material

Kaniu et al. supplementary material 2 Kaniu et al. supplementary material

Kaniu et al. supplementary material 3 Kaniu et al. supplementary material

## Supplementary material

To view supplementary material for this article, please visit https://doi.org/10.1017/ash.2025.41
