# Supplementary material for: Knowledge, attitudes, and practices regarding antimicrobial resistance and antimicrobial stewardship among healthcare workers in outpatient medical centers in Kenya: a qualitative study
Source: Antimicrob Steward Healthc Epidemiol. 2025 May 19;5(1):e113. doi: 10.1017/ash.2025.41 (PMC12089738; doi:10.1017/ash.2025.41)
Supplement: Kaniu et al. supplementary material 1 — Kaniu et al. supplementary material [file S2732494X25000415sup001.docx]

# APPENDIX 1: INTERVIEW GUIDE

**Semi-Structured Interview Guide**

**Section one: Demographic Information**


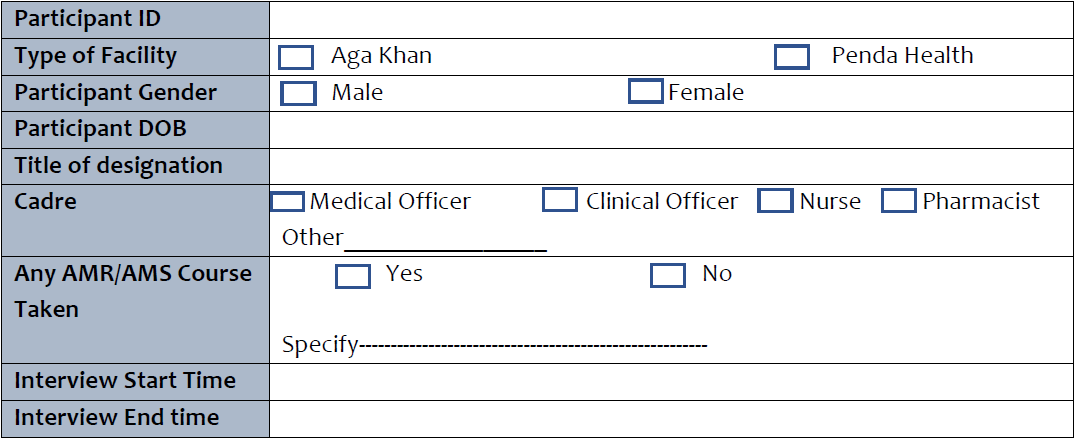
***Section 2:***

***Knowledge***

**Now I would like to ask you about your knowledge on antimicrobial resistance. There is no right or wrong answers**

1. Can you please share with me your knowledge on antimicrobial resistance?
   1. What do you know about it?
   2. What are some of the causes?
2. Do you think it is a problem? Why/Why not?
3. Have you heard of antimicrobial stewardship?
   1. What is it?
   2. Can you describe any activities/elements that make up AMS?
   3. What have you heard about it?
4. Have you ever tried any AMS activities at the clinic?
   1. If yes, what activities have you tried?
   2. Why did you try them?
5. Are there any AMS activities that you would like to implement in the clinic and haven’t
   1. If so what are they, and why haven’t you implemented them?
6. Are there any ongoing AMS activities at the clinic currently?
   1. What kind of activities?
   2. What is your impression of how that is going?

***Prioritization***

**Now I would like to ask you about your thoughts around prioritization of antimicrobial resistance in general. There are no right or wrong answers**

1. Do you think AMR is a problem in your clinic?
   1. What about in Kenya and the world? Is it a problem?
   2. How big a problem is AMR is in your clinic, Kenya and the world?
   3. Why/Why not?
2. Do you think the fight against AMR is important?
   1. How important is it?
   2. Why is it important?
3. How important do you think implementing AMS is at the clinic level?
   1. How important is it?
   2. Why is it important?
4. How much of a priority is AMS in your clinic?
   1. Why is it a priority?
   2. Why is it not a priority?
5. What level of resources would you put into this activity compared to other activities?
   1. Why/why not?
6. Any further suggestions on how we can prioritize AMS at the clinic level?
   1. Who do we need to involve?
   2. How can we involve them?

***Attitudes***

**Now I would like to ask you about general attitudes around antimicrobial resistance at your clinic. There is no right or wrong answers**

1. What do you think about AMS at your clinic?
   1. Would it be easy or difficult and why?
   2. Would it require people with special skills that may not be represented at the clinic?
2. Would you be willing to implement AMS at your clinic if it were possible?
   1. Why/why not
3. What are your impressions about AMS in general?
   1. is it an activity primarily for inpatient or outpatient medicine?
   2. Would it succeed in your setting?
4. What would you suggest is done for AMS to work at your clinic?
