# Supplementary material for: Knowledge, attitudes, and practices regarding antimicrobial resistance and antimicrobial stewardship among healthcare workers in outpatient medical centers in Kenya: a qualitative study
Source: Antimicrob Steward Healthc Epidemiol. 2025 May 19;5(1):e113. doi: 10.1017/ash.2025.41 (PMC12089738; doi:10.1017/ash.2025.41)
Supplement: Kaniu et al. supplementary material 2 — Kaniu et al. supplementary material [file S2732494X25000415sup002.docx]

**APPENDIX 2: Additional quotes from the in-depth interviews**

**Definition on Antimicrobial resistance**

*“My understanding of antimicrobial resistance is…where someone stops responding to antibiotics probably as a result of overuse or underuse of the same. That is what I'd say but they stop responding like they should to a particular treatment.”* KII_023*Medical officer*

**AMS Knowledge gaps**

“*Interviewer: Have you heard of antimicrobial stewardship?*

*Interviewee: No*

*Interviewer: If it is to be implemented, what level of resources would you think would be required?*

*Interviewee: That one I don't know.”* KII_007 Other staff

**Covid-19 exacerbating self-prescribing**

*“It's not like they like [self-prescribing], but you know after COVID, there was the liberal use of specific antibiotic, self-prescription, people just going into any chemist, and buying, probably they have tried several things, including sometimes an antibiotic, and most likely a wrong dose or frequency or duration, so by the time you are seeing them it's like you want to streamline whatever care they have already started, so you are not sure whether to stop the whole thing or guide them on how to finish properly…some it is more warranted but then most of the prescriptions from outside they always have antibiotics.”* KII_001 Medical officer

**Barriers to and needs for AMS implementation**

“*Sometimes in the pharmacy you find that the lower levels of antibiotics are not there, so you will be forced to prescribe what is there. That also can lead to antimicrobial resistance.” KII_023 Medical Officer*

*“You know when you are implementing something there is usually resistance to change and all that. It is not something we can tell you we can do overnight.*” KII_018 Medical Officer

“*Well here I don’t think there’s a reason but maybe there’s no initiative and maybe the main hospital needs to see their level of importance… Because most CMEs are organized from the main hospital so just to create awareness and see the importance of antimicrobial stewardship.” KII_023 Medical Officer*

**Opportunities and ideas**

*“First, people need to be trained fully, for it to sink into the people’s minds… People need to really understand and then from there they can be given time to digest. This is not something you can just wake up and put into the people. We know resistance is something that is happening, but you can't just wake up and put it in people’s mind.”* KII_009 Other staff.

*“There is not much research that has been done out there, especially in the field of antibiotic resistance. I think we need to go into that direction and do more research and give more information, so that when we give it to the policy makers, we can understand what we are talking about.”* KII_011 Other staff.

**Importance and prioritization**

“*The real problem lies with us here at the outpatient setting because as I have said, people try other things and then they come here. So, when we see it is a serious case that is when we send them for inpatient management. So, the root is the outpatient. So, basically, in my opinion antimicrobial stewardship should target the primary healthcare setting..*.” KII_004 Medical Officer.

“*In terms of priority, in this institution we have not put it as one of the major priorities. We have put priorities in other things. If I rate it, I would give it on a scale of 1-10, 2 or 3. It is not a big priority.”* KII_011 Other staff

**Attitudes**

*Interviewer: How willing and receptive would your team be to implement AMS?*

*Interviewee: They would be receptive. We have all the resources we need. So I believe it is just to champion them towards this cause…it is just sensitization and having the right motivation for this.”* KII_015 Medical Officer
